# Antigen-specific memory Th17 cells promote cross-protection against nontypeable *Haemophilus influenzae* after mild influenza A virus infection

**DOI:** 10.1016/j.mucimm.2023.01.007

**Published:** 2023-02-02

**Authors:** Xinyun Zhang, Ying Yang, ShengSen Chen, Wenchao Li, Yong Li, Brian J. Akerley, Linyun Shao, Wenhong Zhang, Hao Shen, Michael C. Abt

**Affiliations:** 1 Department of Infectious Diseases, Shanghai Key Laboratory of Infectious Diseases and Biosafety Emergency Response, National Medical Center for Infectious Diseases, Huashan Hospital, Fudan University, Shanghai, China.; 2 Department of Microbiology, University of Pennsylvania Perelman School of Medicine, Philadelphia, USA.; 3 Department of Endoscopy, Cancer Hospital of the University of Chinese Academy of Sciences (Zhejiang Cancer Hospital), Institute of Cancer and Basic Medicine (IBMC), Chinese Academy of Sciences, Hangzhou, China.; 4 Shanghai Institute of Immunology, Shanghai Jiaotong University, Shanghai, China.; 5 Department of Immunology and Rheumatology, the Affiliated Drum Tower Hospital of Nanjing University Medical School, Nanjing, China.; 6 Department of Cell and Molecular Biology, University of Mississippi Medical Center, Jackson, Mississippi, USA.; 7 National Clinical Research Center for Aging and Medicine, Huashan Hospital, Fudan University, Shanghai, China.; 8 Key Laboratory of Medical Molecular Virology (MOE/MOH), Shanghai Medical College, Fudan University, Shanghai, China.

## Abstract

Secondary bacterial pneumonia after influenza A virus (IAV) infection is the leading cause of hospitalization and death associated with IAV infection worldwide. Nontypeable *Haemophilus influenzae* (NTHi) is one of the most common causes of secondary bacterial pneumonia. Current efforts to develop vaccines against NTHi infection focus on inducing antibodies but are hindered by antigenic diversity among NTHi strains. Therefore, we investigated the contribution of the memory T helper type 17 (Th17) response in protective immunity against IAV/NTHi coinfection. We observed that even a mild IAV infection impaired the NTHi-specific Th17 response and increased morbidity and mortality compared with NTHi monoinfected mice. However, pre-existing memory NTHi-specific Th17 cells induced by a previous NTHi infection overcame IAV-driven Th17 inhibition and were cross-protective against different NTHi strains. Last, mice immunized with a NTHi protein that induced a strong Th17 memory response were broadly protected against diverse NTHi strains after challenge with coinfection. These results indicate that vaccination that limits IAV infection to mild disease may be insufficient to eliminate the risk of a lethal secondary bacterial pneumonia. However, NTHi-specific memory Th17 cells provide serotype-independent protection despite an ongoing IAV infection and demonstrate the advantage of developing broadly protective Th17-inducing vaccines against secondary bacterial pneumonia.

## INTRODUCTION

Each year, 25,000–50,000 deaths worldwide are attributed to infection by influenza virus or influenza virus-related complications^1^. Most patients with influenza A virus (IAV) infection have relatively mild, self-limited illnesses^2–6^, partly as a result of annual vaccination. Severe disease and deaths associated with influenza virus infection are most often due to secondary bacterial infections^7–9^, which are even more closely correlated with mortality during flu pandemics^8,10,11^. Despite the widespread use of vaccines and availability of antibiotics, secondary bacterial infections remain a leading threat in both influenza virus epidemics and pandemics^12,13^. There is limited clinical evidence that demonstrates influenza virus infection that causes severe symptoms exclusively leads to secondary bacterial pneumonia. Previous animal studies modeling coinfection have only used an infectious dose of influenza virus that causes severe symptoms as a single infection^14–16^. The current dogma is that an influenza virus infection that causes severe initial lung damage is needed to overwhelm the host’s defenses and predispose individuals to a secondary bacterial infection causing lethality, whereas a mild IAV infection is not sufficient to impair host antibacterial defenses. This study sought to address whether individuals experiencing mild influenza symptoms are still at risk of developing severe bacterial pneumonia using a low dose influenza virus/bacterial coinfection model in mice.

*Haemophilus influenzae,* a Gram-negative coccobacillus bacterium, is one of the most common pathogens that causes secondary bacterial pneumonia after influenza virus infection^17–19^. *H. influenzae* is classified into two groups, typeable and nontypeable^20^. Typeable *H. influenzae* possesses polysaccharide capsules, whereas nontypeable *H. influenzae* (NTHi) lacks a capsule^21^. Current highly effective conjugate vaccines are based on antibody responses targeting the polysaccharide capsule that is expressed by *H. influenzae* type b^22^; however, these vaccines have no effect on NTHi infection^23,24^. Thus, NTHi has become the primary cause of *H. influenzae* diseases in populations vaccinated against *H. influenzae* type b^25^. NTHi colonizes the nasopharynx of up to 75% of healthy adults^26^. Certain conditions, such as immunosuppression and viral infection, compromise the containment of NTHi in the nasopharynx, allowing it to spread to other sites where it causes a spectrum of diseases, including otitis media, community-acquired pneumonia, and exacerbation of chronic obstructive pulmonary disease^26–28^. Antibiotic use to treat NTHi infections is common but not always effective and contributes to the global burden of bacterial antibiotic resistance^29–31^. Thus, a better understanding of immune mechanisms that can broadly protect against antigenically diverse NTHi strains is essential to develop effective treatments and preventive measures.

NTHi vaccines under development focus on antibody responses that target bacterial surface proteins and lipooligosaccharide antigens^26,32^. However, NTHi vaccines containing NTHi surface protein had limited efficacy to augment pulmonary bacterial clearance and provide protection in mouse studies^14,33^ and human clinical trials^23,34^ despite inducing high NTHi-specific antibody titers. Progress in developing NTHi vaccines has been hindered by genetic and antigenic diversity of NTHi strains and there is no universally approved vaccine against NTHi^26,35,36^. Although effective at limiting severity against influenza virus single infection, influenza virus vaccines confer very limited protection against viral and bacterial coinfection^37–40^. Therefore, even an influenza infection that causes mild symptoms may be potentially lethal if combined with a secondary bacterial infection. Thus, there is an urgent need to investigate immune mechanisms other than antibodies that can provide broad and effective protection against secondary bacterial infections.

An alternative to antibody-based immunity against IAV/NTHi coinfection involves T cell-mediated mechanisms. Cluster of differentiation (CD)4^+^ T helper 17 (Th17) cells are increasingly being recognized as key mediators of protection against airway infection by respiratory pathogens^41–43^. The production of interleukin (IL)-17A mediates many Th17 effector functions at mucosal surfaces, resulting in increased recruitment of macrophages and neutrophils and enhanced cytotoxicity and phagocytosis^44–46^. IL-17A production is essential for defense against several respiratory mucosal pathogens, including *Mycoplasma pneumoniae*, *Streptococcus pneumoniae*, *Klebsiella pneumoniae*, and *Bordetella pertussis*^41–43^. Our recent work showed that the recall responses from memory Th17 cells in NTHi immunized mice drive antibody-independent protection against pulmonary infection with heterologous NTHi strains through recognition of conserved cytosolic and membrane-associated antigens^47^. Similarly, intranasal immunization with NTHi outer membrane vesicles stimulated Th17 memory formation that subsequently protected against heterologous strains^48^. However, in the murine models of IAV and *Streptococcus pneumoniae* coinfection, a preceding influenza virus infection decreases the secretion of IL-17, leading to impaired immunity against *S. pneumoniae*^16,18^. Similar observations were reported for influenza virus and *S. aureus* coinfection, in which primary influenza virus infection led to a decrease in IL-17 production in response to *S. aureus* coinfection^49^. Furthermore, the Th17 cell lineage is plastic and Th17 differentiated cells can switch to other Th subsets in the presence of Th1 or Th2-inducing cytokines^50^. Th17 responses are broadly protective against pulmonary bacterial single infection and decreased Th17 responses are associated with susceptibility to secondary bacterial infection after IAV infection^16,18,47,48^. These reports raise uncertainty as to whether a vaccine designed to elicit bacterial-specific Th17 memory can overcome viral-driven Th17 inhibition and mount a protective Th17 recall response against subsequent NTHi infection in the context of a mild IAV infection.

In this study, we first tested primary Th17 responses in the lungs of IAV/NTHi coinfected mice using a low dose of IAV to more closely replicate a secondary bacterial infection in an individual experiencing asymptomatic/mild influenza virus infection and establish that secondary bacterial infections can be lethal even when the primary viral infection does not cause severe symptoms. Further, it is unclear whether recall responses by preformed memory Th17 cells are inhibited by IAV infection. Therefore, we investigated if pre-existing NTHi-specific memory Th17 cells can rescue the Th17 response and convey sufficient cross-protection against coinfection. Through these studies, we found that NTHi-specific Th17 responses during a primary coinfection were inhibited even at a very low dose of IAV infection; however, established NTHi-specific memory Th17 cells overcame IAV-driven inhibition of Th17 responses and conveyed cross-protection against secondary NTHi infection after IAV infection by mounting a protective Th17 recall response. Collectively, our results are an important step toward the development of a broadly protective vaccine against NTHi and IAV/NTHi coinfection.

## RESULTS

### Mice infected with a low dose of IAV are susceptible to secondary NTHi infection

**Fig. 1:**
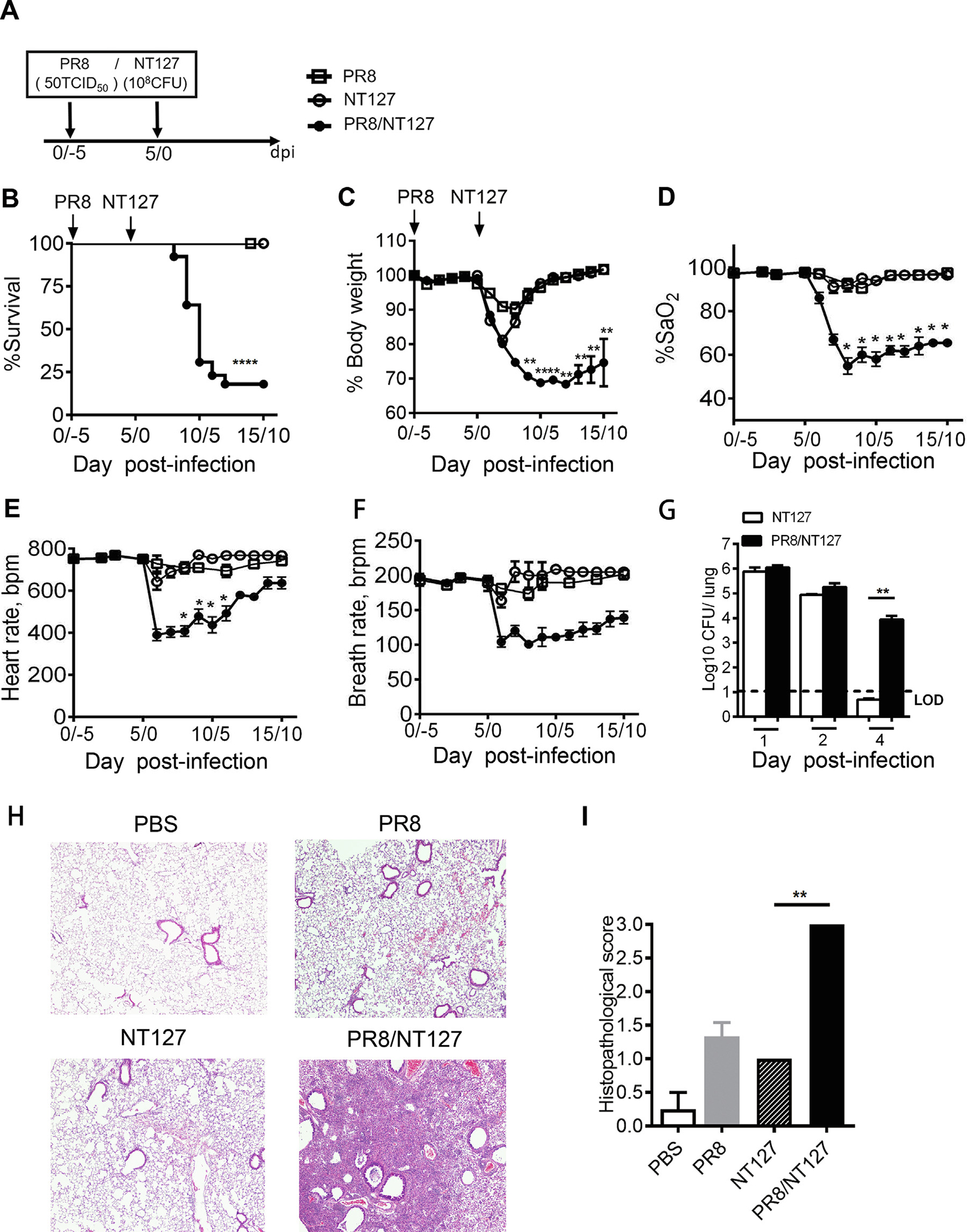
Mice with mild IAV infection are still susceptible to secondary infection with NTHi. (A) Experimental design. Low dose PR8 (50TCID_50_)-infected mice were infected with NTHi (NT127 strain) (10^8^ CFU) at day 5 and monitored daily; (B) Survival rates of PR8-infected mice, NT127-infected mice, and PR8/NT127 coinfected mice; (C–F) Body weight loss and lung physiological parameters were measured including: (D) arterial oxygen saturation (%); (E) heart rate (bpm); and (F) breath rate (brpm); (G) Bacterial load in lung homogenates on 1, 2, and 4 days after NT127 infection; (H) Representative slides and; (I) histopathological scores of H&E-stained lung sections were collected on day 7 after NT127 infection. PBS control mice (D0), NT127-infected (D7) mice, PR8-infected mice (D12), PR8 and NT127 coinfected mice (D12/7) in 4X magnification micrographs of original section. Results are representative of at least three independent experiments with 9–10 mice in each group. Data displayed as mean ± SEM. ****p < 0.0001; ***p < 0.001; **p < 0.01; *p < 0.05. bpm = beats per minute; brpm = breath rates per minute; CFU = colony forming units; H&E = hematoxylin and eosin; IAV = influenza A virus; LOD = limit of detection; NTHi = nontypeable *H. influenza*; PBS = phosphate-buffered saline; PR8 = influenza A/Puerto Rico/8/34/H1N1 virus; SEM = standard error of mean; TCID_50_ = 50% tissue culture infectious dose.

Most influenza virus infections are mild and self-limiting with the host normally fully recovering within 2 weeks of infection^3–6^. Serious complications associated with influenza virus infection are not due to primary viral pneumonia but are instead driven by secondary bacterial pneumonia^9^. We previously established that IAV infection increases host susceptibility to subsequent NTHi infection using a dose [200 50% tissue culture infectious dose (TCID_50_)] of IAV that causes severe disease as a single infection^51^. To test whether individuals with asymptomatic/mild flu are susceptible to subsequent bacterial infection, we infected mice intranasally (i.n.) with a low dose (50 TCID_50_) of influenza A/Puerto Rico/8/34/H1N1 virus (PR8), followed by intranasal inoculation with NTHi (NT127 strain) 5 days later (Fig. 1A). The mice infected with PR8 or NT127 alone all survived and exhibited disease classified as mild^52^, defined by transient weight loss of approximately 10% and no other sign of morbidity (oxygen saturation, heart rate, and breath rate) (Figs. 1C–F and Supplementary Fig. S1B). In contrast, PR8/NT127 coinfected mice had a very low survival rate of ~18% (Fig. 1B). Coinfection with PR8 and NT127 caused severe disease with substantial weight loss (Fig. 1C) and a significant decrease in oxygen saturation (Fig. 1D), heart rate (Fig. 1E), and breath rate (Fig. 1F), leading to 82% mortality after bacterial infection by day 5 (Fig. 1B). Next, the mice were infected with 50 TCID_50_ of PR8, followed by titrating doses of NT127 from 10^6^-10^8^ colony forming units (CFUs) and assessed for morbidity and mortality. At the low dose of IAV infection that caused little signs of morbidity, coinfection with 10^7^ and 10^8^ CFU of NT127 resulted in 46% and 80% mortality, respectively (Supplementary Fig. S1A).

To further evaluate host susceptibility to secondary bacterial infection after low dose IAV infection, bacterial loads and lung pathology in coinfected mice were determined over the course of NT127 bacterial infection. During the first 2 days after the NT127 infection, similar bacterial loads were detected in the lungs of NT127-infected and PR8/NT127 coinfected mice (Fig. 1G). By day 4, after NT127 infection, the animals infected with NT127 alone had undetectable bacterial burdens in the lungs, whereas coinfected mice had ~10^4^ CFU of NT127 in the lungs (Fig. 1F). Histology showed that coinfected mice displayed more severe pathology and inflammation in their lungs than mice infected with NT127 alone at day 7 after the NT127 challenge (Figs. 1H and 1I). Taken together, these results demonstrate that previous IAV infection, even at dose of 50 TCID_50_ that causes mild disease as a single infection, still increases host susceptibility to secondary NTHi infection.

### NTHi-specific Th17 responses are inhibited during IAV/NTHi coinfection

**Fig. 2:**
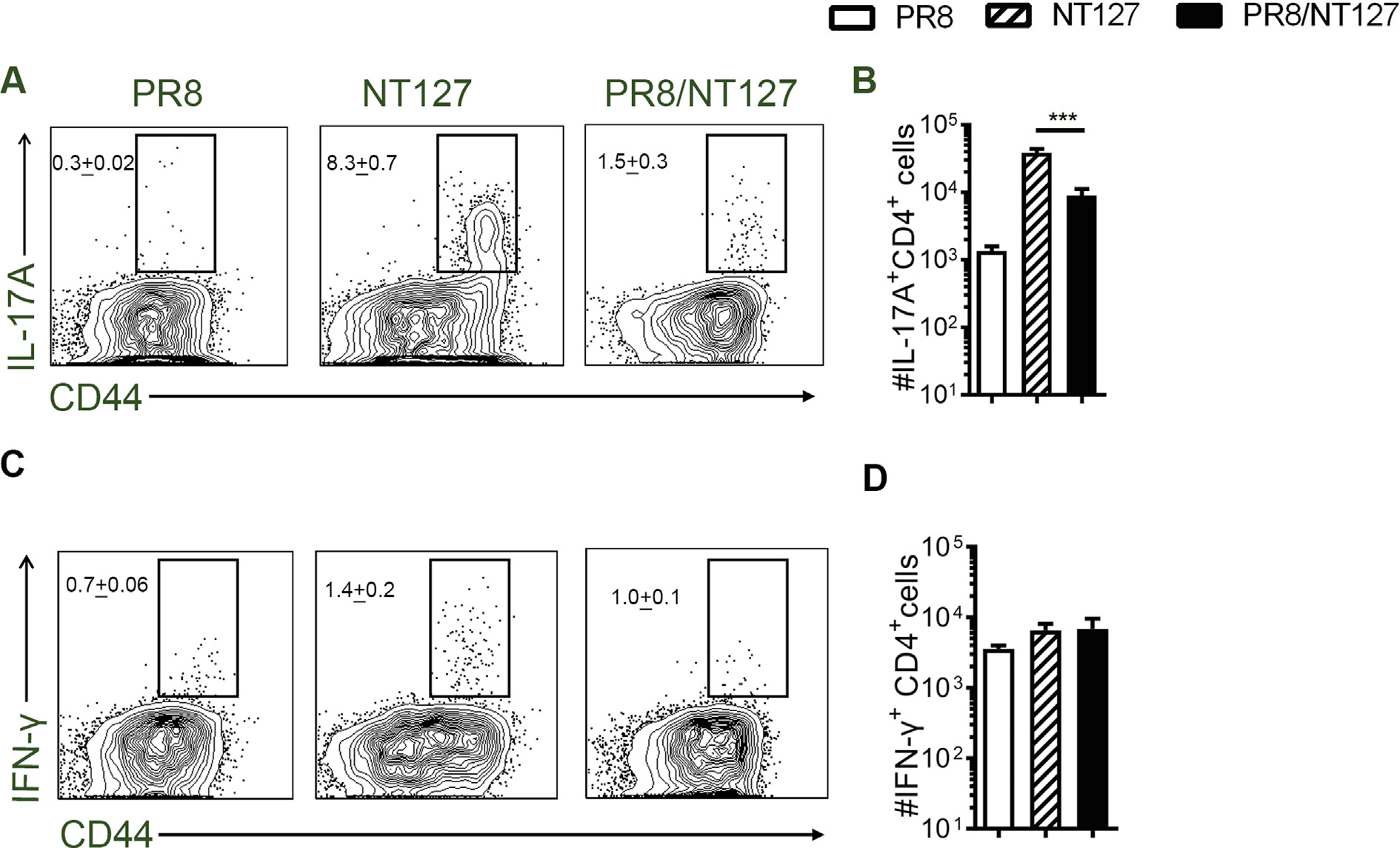
Influenza virus infection suppresses NTHi-specific Th17 response during coinfection in the lung. (A–D) Cells isolated from the lung of mice infected with PR8 or NT127 alone, and mice coinfected with PR8/NT127 at day 7 after NT127 infection were *ex vivo* stimulated with heat-killed (HK) NT127 and IL-17A^+^ CD4^+^, IFN-γ^+^ CD4^+^ T cells per lung were quantified by FACS: (A) Frequency and; (B) absolute number of pulmonary IL-17^+^ CD4^+^ T cells; (C) frequency and; (D) absolute number of IFN-γ^+^ CD4^+^ T cells. FACS plots gated on CD4^+^ CD8^neg^ cells. Data are expressed as the mean value ± SEM of 5–10 mice/group, representative of three independent experiments. ***p < 0.001; **p < 0.01; *p < 0.05.

We next sought to determine the immune response after IAV and NTHi coinfection (Fig. 1A). Elevated bacterial burdens in coinfected mice suggest an impaired immune response to infection (Fig. 1G). Since Th17 cells are critical for host defense against bacterial pneumonia^47^, we examined the Th17 response during the IAV/NTHi coinfection. Using our IAV/NTHi bacteria coinfection system (Fig. 1A), singly infected and coinfected mice were sacrificed at day 7 after the NT127 infection and assessed for NT127-specific Th17 responses in the lung by intracellular cytokine staining (ICS) and flow cytometry (Supplementary Fig. S2, Supplementary Table S1). NT127-infected mice exhibited a strong NT127-specific Th17 responses (8.3% IL-17^+^ CD4^+^ T cells) in the lungs, whereas the surviving PR8/NT127 coinfected mice had significantly lower frequencies (1.5%) and lower total numbers of NT127-specific Th17 cells (IL-17^+^ CD4^+^ T cells) in the lungs (Figs. 2A and 2B). A low number of NT127-specific Th1 cells [interferon (IFN)-γ^+^ CD4^+^ T cells] were detected in the lungs of both NT127-infected mice and coinfected mice (Figs. 2C and 2D). Similar results were observed when cells from infected lungs were stimulated *in vitro* with phorbol myristate acetate/ionomycin (PMA/I) to activate total Th17 responses (Supplementary Fig. S3A). Likewise, there were fewer total IL-17^+^ CD4^+^ T cells in the lung of coinfected mice than NT127 singly infected mice after PMA/I stimulation (Supplementary Fig. S3B). However, in coinfected mice, there were stronger total Th1 responses in the lung compared with NT127 singly infected mice (Supplementary Figs. S3C and S3D). Thus, the primary T cell response to NT127 infection of the lung is a strong Th17 response with a small population of Th1 responses. However, the primary Th17 response is much lower in the lungs of PR8/NT127 coinfected mice. Together, these results show that protective Th17 responses mounted during NTHi single infection are inhibited in the lungs during IAV/NTHi coinfection, which may contribute to host vulnerability to secondary NTHi infection.

### Prior NTHi exposure confers protection against IAV/NTHi coinfection

**Fig. 3:**
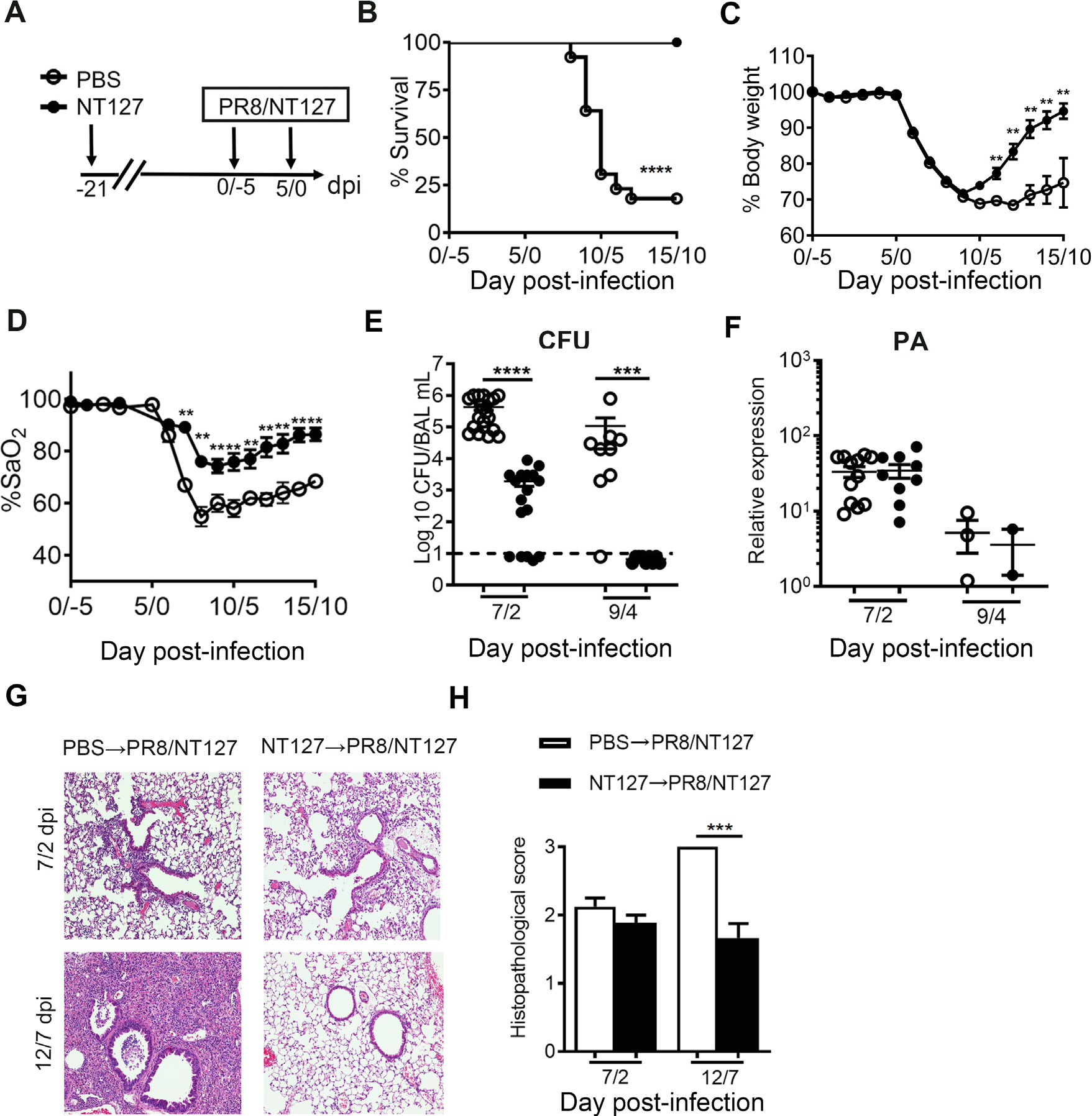
Prior NTHi exposure confers protection against IAV/NTHi coinfection. (A) Experimental schema of immunization strategy. Mice were inoculated with live NT127 (10^8^ CFU) for 21 days and then were challenged with PR8/NT127 coinfection; (B) survival rate; (C) body weight loss and; (D) arterial oxygen saturation rate; (E) bacterial load in bronchoalveolar lavage (BAL) fluid at 2 and 4 days after NT127 infection; (F) relative expression of PR8 polymerase (PA) in lung homogenate at 2 and 4 days after NT127 infection; (G) representative images and; (H) histopathological scores of airways at 2 and 7 days after NT127 infection with H&E staining. (B-E) *n* = 5–19 mice per group; (F-H) *n* = 2–5 mice per group. Data were expressed as the mean value ± SEM, representative of three independent experiments. ****p < 0.0001; ***p < 0.001; **p < 0.01; *p < 0.05.

Our previous results demonstrate that established Th17 memory protects against single NTHi infection by mounting a recall Th17 response^47^. However, because IAV infection inhibits the primary Th17 response (Fig. 2) and differentiated Th17 cells exhibit plasticity in the context of a Th1 or Th2-driving microenvironment and are able to switch to other Th subsets^50^, we hypothesized that the recall response by memory Th17 cells would also be inhibited by IAV infection and would not protect mice against subsequent IAV/NTHi coinfection. To determine whether a Th17 memory response induced by prior NTHi exposure was impaired in providing protection against subsequent NTHi infection in the context of an IAV-induced Th1 environment, mice were infected with NT127 and 3 weeks later, challenged with PR8/NT127 coinfection (Fig. 3A). We have previously reported that 21 days after infection is sufficient time to generate stable tissue-resident memory Th17 cell population, predominantly in the lung^47^. Surprisingly, mice that were exposed to prior NT127 infection (NT127-immune) were protected against subsequent coinfection with 100% survival, whereas mice inoculated with phosphate-buffered saline (PBS) only (unimmune) exhibited only 20% survival (Fig. 3B), thus refuting our hypothesis. Overall, the NT127-immune mice also lost less body weight (Fig. 3C), maintained higher levels of arterial oxygen saturation (SaO_2_) (Fig. 3D), and recovered body weight and SaO_2_ levels faster than unimmune mice (Figs. 3C and 3D). The bacterial loads in the bronchoalveolar lavage (BAL) fluid of NT127-immune mice were significantly lower than in unimmunized mice (Fig. 3E). NT127-immune mice cleared bacteria in the lungs by day 4 after NT127 infection, whereas unimmune mice maintained high levels of bacteria (~10^5^ CFU), with most succumbing between 4–7 days (Figs. 3B and 3E). There was no difference in the amount of viral mRNA for polymerase (PA) in the lungs between unimmunized and immune mice on day 2 and 4 post NT127 infection (Fig. 3F). Histology showed that NT127-immune mice had fewer infiltrating inflammatory cells and less tissue damage (Fig. 3G), resulting in significantly reduced histopathology scores (Fig. 3H) compared with unimmunized mice. Together, these results indicate that immune memory induced by prior lung NTHi infection is sufficient to promote bacterial clearance, limit tissue inflammation, and prevent mortality during IAV/NTHi coinfection.

### Memory T cells mount strong NTHi-specific Th17 recall responses during coinfection

**Fig. 4:**
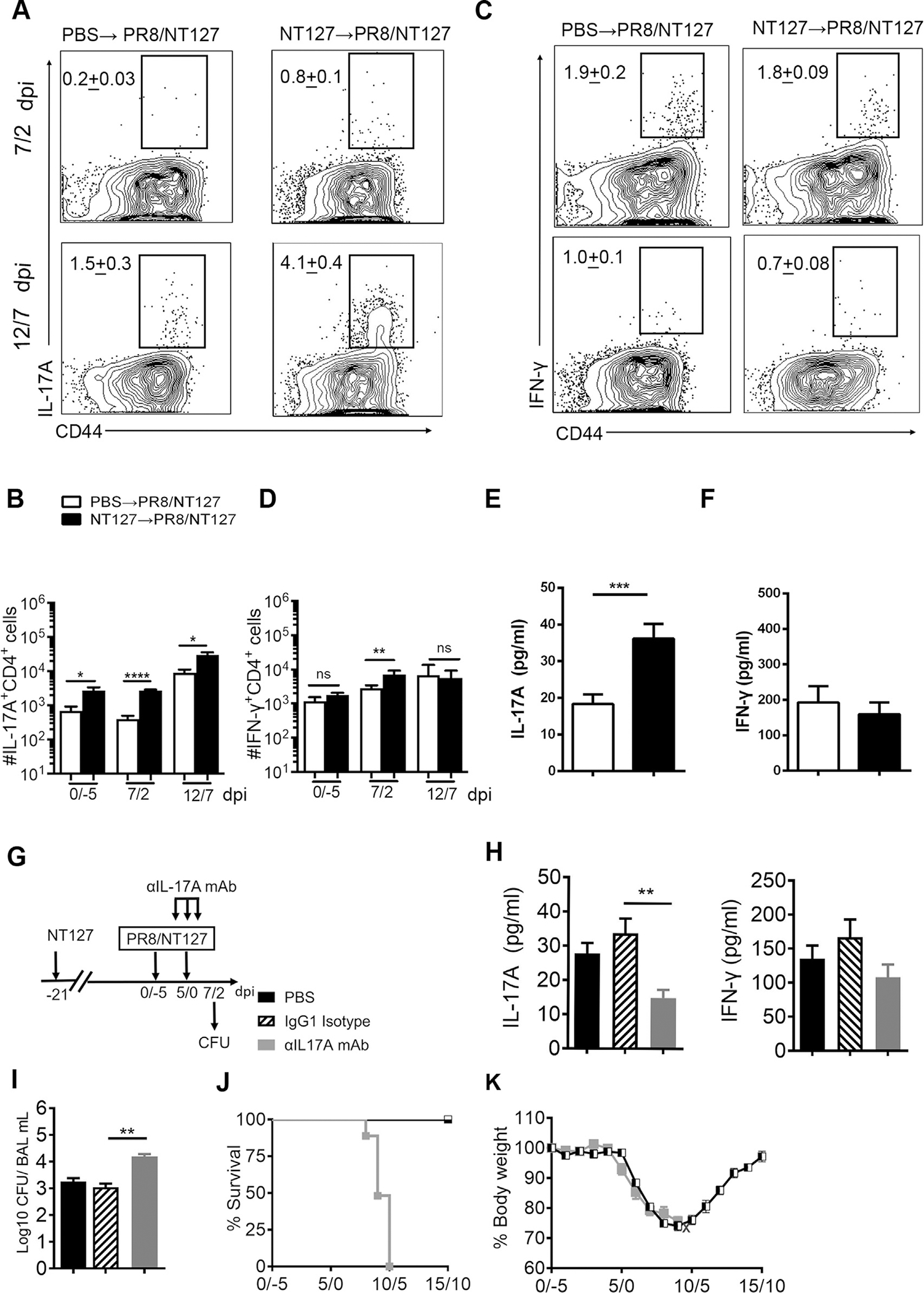
Memory T cells mount high NTHi-specific Th17 response during coinfection in the lung. (A) Frequency and; (B) absolute cell counts of IL-17A^+^ CD4^+^ T cells isolated from lungs at day 0/−5 before coinfection (day 21 after immunization), day 7/2 and 12/7 after PR8/NT127 challenge and stimulated with HK-NT127; (C) Frequency and; (D) absolute cell counts of IFN-γ^+^ CD4^+^ T cells isolated from lung on day 0/−5, 7/2 and 12/7 and stimulated with HK-NT127 after last NT127 infection. FACS plots gated on CD4^+^ CD8^neg^ cells; (E) The concentration of IL-17A and; (F) IFN-γ in BAL fluid on day 2 after NT127 challenge; (G) Experimental schema of *in vivo* IL-17A neutralization. Mice were infected by NT127 and then were challenged by NT127 at day 5 following PR8 infection 3 weeks after prior NT127 infection. Mice were treated with anti-IL-17A antibody, IgG isotype control or PBS on Day −1,0,1 after NT127 challenge; (H) Concentrations of IL-17A, IFN-γ were determined by ELISA, and; (I) bacterial load in BAL fluid, 2 days after NT127 challenge. (J) Survival rate and (K) body weight loss of NT127 immunized mice that were treated with IL-17A neutralizing antibody and IgG isotype antibody. Error bars represent SEM. Data are expressed as the mean value ± SEM of 3–5 mice/group, representative of three independent experiments. ****p < 0.0001; ***p < 0.001; **p < 0.01; *p < 0.05; ns, not significant.

To better understand the immune memory properties that protect against IAV/NTHi coinfection, we further investigated the recall Th17 responses throughout the course of coinfection in NTHi immunized and nonimmunized mice. On day 7/2 post PR8/NT127 coinfection, there were significantly more NT127-specific IL-17^+^ CD4^+^ T cells in NT127-immune mice (0.8%) than in unimmunized mice (0.2%) (Fig. 4A), indicating a recall response in NT127-immune mice. By day 12/7 after PR8/NT127 coinfection, NT127-immune mice exhibited a robust Th17 response (4.1% IL-17^+^ CD4^+^ T cells), whereas Th17 responses in nonimmune control mice remained weak (1.5% IL-17^+^ CD4^+^ T cells) (Fig. 4A). Consistent with the frequency of Th17 cells, there were greater total numbers of IL-17A^+^ CD4^+^ T cells in the lung of NT127-immune mice than control mice (Fig. 4B). Furthermore, there were higher levels of IL-17A protein in the BAL fluid of NT127-immune compared with the control mice (Fig. 4E). In contrast, there was no significant difference in the frequency or absolute number of pulmonary NT127-specific Th1 cells at day 12/7 post coinfection (Figs. 4C and 4D) and no difference in IFN-γ protein in the BAL fluid (Fig. 4F) during coinfection between these two groups. To further investigate if protective immunity against PR8/NT127 coinfection requires NTHi-specificity or if any population of lung resident memory Th17 cells can convey protection, we intranasally immunized mice with *S. pneumoniae* strain TIGR4 (T4, serotype 4), which can induce T4-specific Th17 responses in the lung^53^, and then challenged the immunized mice with PR8/NT127 at day 21 post immunization. On day 21 post immunization, we stimulated lymphocytes from lungs with HK-T4 or HK-NT127. HK-T4 but not HK-NT127 stimulation induced a population of IL-17^+^ CD4^+^ T cells demonstrating that there were memory Th17 responses specific to T4 but no memory Th17 responses reactive to NT127 (Supplementary Figs. S4A and S4B). Next, we tested the protection conferred by T4-specific Th17 memory cells against PR8/NT127 challenge. T4-immune and PBS group had comparable mortality rate after PR8/NT127 coinfection (Supplementary Fig. S4C), indicating T4-specific Th17 memory cells have no protective capacity against PR8/NT127 coinfection. These results show that NTHi-specific memory Th17 cells induced by prior NTHi infection can mount a protective Th17 recall effector response against secondary bacterial infection despite the presence of an ongoing influenza virus infection, correlating with protection in these mice.

We next determined the role of the Th17 cell canonical effector cytokine, IL-17A, in the protection against coinfection in NT127-immune mice by *in vivo* neutralization of IL-17A on day 4/−1, 5/0, and 6/1 during PR8/NT127 challenge (Fig. 4G). As expected, mice treated with PBS or isotype control immunoglobulin (Ig)G-treated groups had similar levels of IL-17A and IFN-γ in the BAL fluid and comparable bacterial loads (Fig. 4H). In contrast, there were significantly low levels of IL-17A in the BAL fluid of anti-IL-17A treated mice, whereas IFN-γ levels were not affected (Fig. 4H). The bacterial loads in the anti-IL-17A-treated mice were nearly 10-fold higher than in the PBS- and isotype-treated control mice at day 7/2 post PR8/NT127 coinfection (Fig. 4I). Furthermore, isotype-treated control mice started to regain body weight at day 10/5 and fully recovered to their original body weight at day 15/10 post coinfection, whereas the anti-IL-17A-treated mice all succumbed to coinfection (Figs. 4J and 4K). These data show that IL-17A blockade abrogates protective immunity against secondary bacterial rechallenge.

### Prior NTHi infection induced Th17-mediated cross-protection against subsequent IAV/NTHi coinfection

**Fig. 5:**
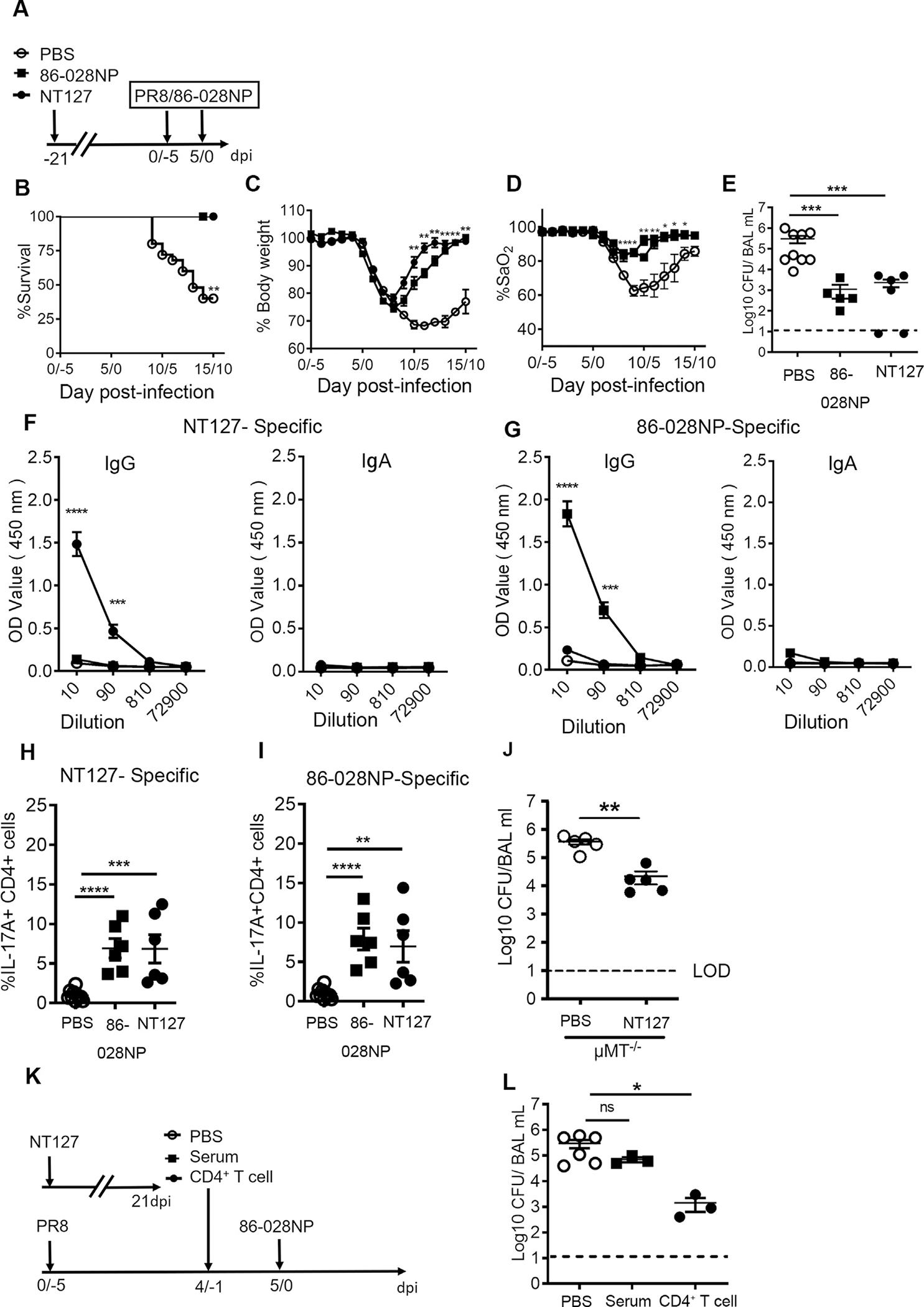
NTHi-specific memory Th17 cells provide broad protection independent of antibody response. (A) Experimental design. Mice were infected with NT127 or 86-028NP and 21 days later were challenged by PR8 and 86-028NP coinfection. Heterologous infected group (NT127→PR8/86-028NP), homologous infected group (86-028NP→PR8/86-028NP), control group (PBS→PR8/86-028NP); (B) survival rate; (C) body weight loss and; (D) arterial oxygen saturation; (E) bacterial load in BAL fluid 2 days after 86-028NP infection; (F) NT127-specific and; (G) 86-028NP-specific IgG and IgA level in the BAL fluid at day 7 after 86-028NP infection; (H–I) Frequency of IL-17A producing CD4^+^ T cells in the lung at day 7 after 86-028NP infection. Cells isolated from the lung were *ex vivo* stimulated with HK- (H) NT127; or (I) 86-028NP and assessed from IL-17A production; (J) μMT^−/−^ mice were infected intranasally with PBS or NT127 and 21 days later were challenge with PR8/86-028NP. Bacterial loads at day 2 after 86-028NP challenge in the lung of PBS and NT127 immunized mice; (K) Mice were immunized with NT127, and CD4^+^ T cells and serum were collected at day 21 after immunization. The CD4^+^ T cells and serum were transferred to PR8/86-028NP coinfected mice by tail vein injection one day before 86-028NP infection; (L) Bacterial loads in the recipient mice were tested at day 2 after 86-028NP infection. Mice transferred with PBS were included as controls. Error bars represent SEM. Data pooled from three experiments (*n* = 5–10 mice per group). ****p < 0.0001; ***p < 0.001; **p < 0.01; *p < 0.05.

To test the sufficiency of memory Th17 cells in protection against subsequent coinfection and if cross-protection against IAV/NTHi coinfection can be induced by prior NTHi infection, we immunized mice with NT127 or a heterologous NTHi strain (86-028NP) and 3 weeks later, challenged immunized mice with PR8 and 86-028NP (Fig. 5A). NT127-immune mice were fully protected against subsequent PR8/86-028NP challenge, while unimmunized group exhibited 40% survival (Fig. 5B). NT127- and 86-028NP-immune mice regained body weight (Fig. 5C) and pulmonary function (SaO_2_) (Fig. 5D) at a similar rate and had similarly significantly decreased bacterial loads in the BAL fluid relative to unimmunized mice (Fig. 5E). These results indicate that prior NT127 infection induced cross-protection against PR8/86-028NP challenge and, importantly, to a level that is comparable to homologous protection in 86-028NP-immune mice.

To examine the immune mechanism of cross-protection, we analyzed antibody and Th17 responses in NT127- and 86-028NP-immune mice challenged with PR8/86-028NP on day 12/7 post challenge. NT127-specific IgG was detected in the BAL fluid of NT127-immune mice but not in 86-028NP-immune mice and *vice versa* (Figs. 5F and 5G). NT127 and 86-028NP specific IgA was undetectable in the BAL fluid of either NT127- or 86-028NP-immunized mice (Figs. 5F and 5G). Thus, IgG responses are highly strain-specific and do not cross-react to heterologous strains. In contrast, IL-17^+^ CD4^+^ T cells were detected by ICS after stimulation with heat-killed (HK) NT127 (Fig. 5H) or 86-028NP (Fig. 5I) in the lung of NT127- and 86-028NP-immune mice, indicating cross-reactive Th17 cells in the lungs that respond to both NT127 and 86-028NP bacterial strains (Figs. 5H and 5I). To further exclude the effect of antibody responses, μMT^−/−^ mice were infected i.n. with NT127 and 21 days later, were challenge with PR8/86-028NP. NT127-immunized mice had significantly reduced bacterial titers (17-fold) in the BAL fluid at day 2 after 86-028NP challenge than unimmunized mice (Fig. 5J). These data indicate that previous bacterial exposure induces cross-protective memory T cells against subsequent coinfection, independent of antibodies. To further test the contribution of CD4^+^ T cells in cross-protection against coinfection, the sera or CD4^+^ T cells isolated from NT127-immunized mice were intravenously transferred to PR8/86-028NP coinfected mice 1 day before 86-028NP infection (Fig. 5K). The mice receiving PBS or serum had comparable high bacterial loads in the BAL fluid at day 2 after 86-028NP infection, whereas mice receiving adoptively transferred CD4^+^ T cells had 2-log lower bacterial burdens (Fig. 5L). These results show that the memory Th17 response induced by prior bacterial infection, but not antibody responses, can convey broad protection against subsequent NTHi infection even in the context of an influenza virus coinfection.

Th17 cells can promote defense against respiratory pathogens in an antibody-independent manner through the recruitment and activation of innate immune cells^54,55^. Therefore, we analyzed major inflammatory cells [neutrophils, alveolar macrophages, and antigen-presenting cells (APCs)] in the lung on day 7/2 after PR8/86-028NP coinfection after NT127 immunization by flow cytometry (Supplementary Fig. S5A). There was no significant difference in lung neutrophil and APC (CD11c^+^CD24^+^-CD64^+^Siglec-F^neg^) infiltration between unimmunized mice and immunized mice (Supplementary Figs. S5B and S5F), but the total numbers of alveolar macrophages were significantly elevated in the lungs of immunized mice (Supplementary Fig. S5D) compared with unimmunized mice. Furthermore, major histocompatibility complex (MHC) II expression on all these inflammatory cells was elevated in immunized mice than in unimmunized mice (Supplementary Figs. S5C, S5E, and S5G). Consistent with elevated innate immune cell activation, the recall Th17 responses at day 7/2 after infection in the lung were significantly increased in the immunized mice compared with unimmunized mice (Supplementary Fig. S5H). These data suggest that rapid activation of memory Th17 cells leads to enhanced innate immune cell activity early after re-infection that leads to improved pathogen clearance from the lung.

### Immunization with a conserved Th17 antigen provided cross-protection against IAV/NTHi coinfection

**Fig. 6:**
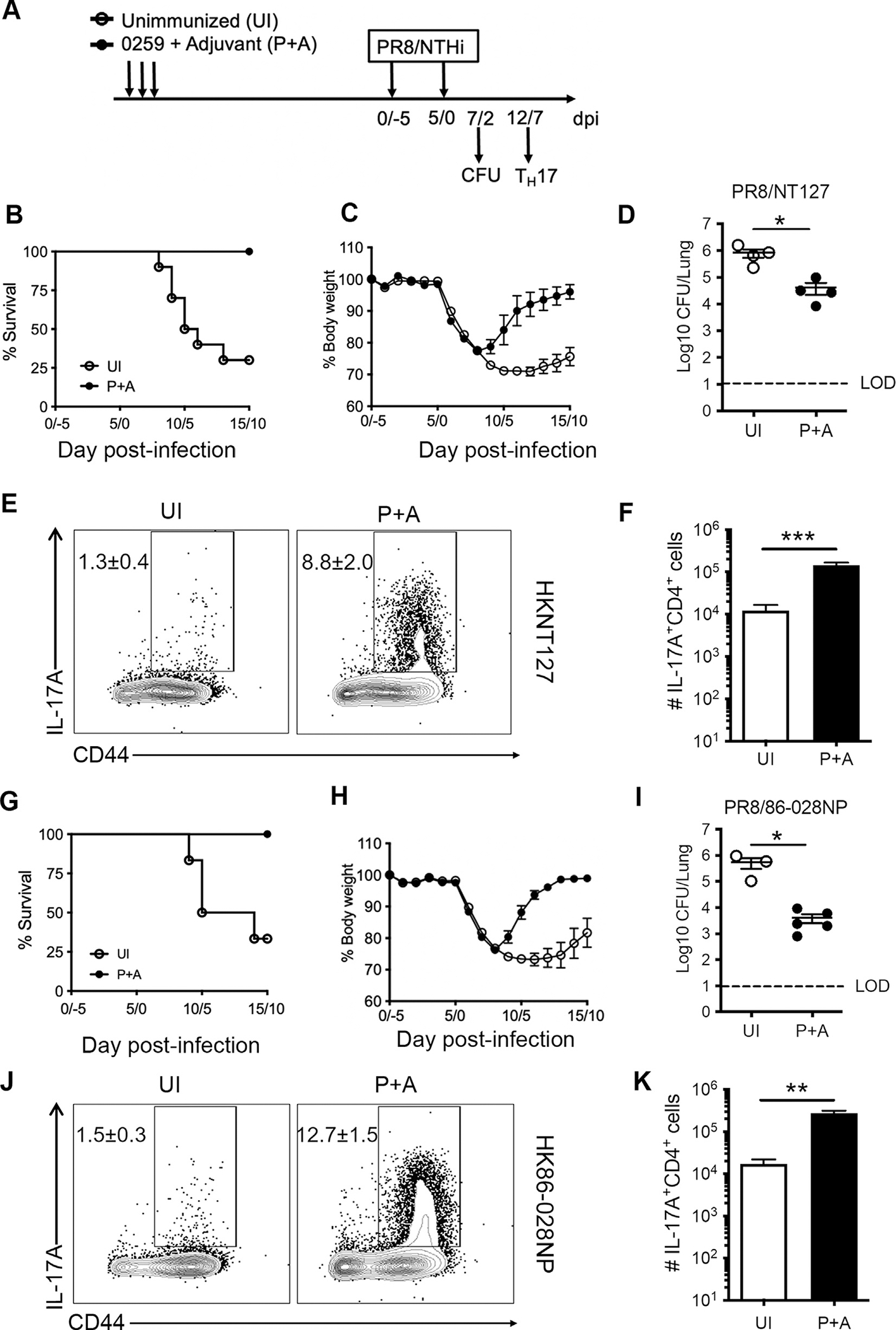
Immunization with a Th17-inducing NTHi-derived antigen induces broad protection against coinfection. (A) Experimental design. B6 mice were immunized intranasally with purified protein 0259 plus the curdlan adjuvant (P+A), three times at one-week intervals. Unimmunized (UI) mice were included as control. Three weeks after final immunization, these mice were challenged intranasally by PR8 and NTHi; (B) survival rate; (C) body weight loss (*n* = 5–10 mice per group), and; (D) bacterial loads on day 2 from lung homogenate (*n* = 4 mice per group); (E) Frequency of IL-17^+^ CD4^+^ cells and; (F) absolute number of pulmonary IL-17^+^ CD4^+^ in cells isolated from the lung on day 7 after homogenous NT127 infection and *ex vivo* stimulated with HK-NT127 (*n* = 5 mice per group); (G) survival rate; (H) body weight loss (*n* = 5–6 mice per group), and; (I) bacterial loads on day 2 from lung homogenate (*n* = 3–5 mice per group); (J) Frequency of IL-17^+^ CD4^+^ cells and; (K) absolute number of pulmonary IL-17^+^ CD4^+^ in cells isolated from the lung on day 7 after heterologous 86-028NP infection and *ex vivo* stimulated with HK-86-028NP (*n* = 3–5 mice per group). Data are representative of two independent experiments. Error bars = means ± SEM. ****p < 0.0001; ***p < 0.001; **p < 0.01; *p < 0.05; ns, not significant.

Our data demonstrate pre-existing Th17 memory cells specific for NTHi can protect mice from IAV/NTHi coinfection driven morbidity and mortality. We have previously identified a conserved NTHi protein antigen (0259) that induces a strong Th17 response and provides protection against pulmonary NTHi single infection^47^. To determine whether immunization with the protein 0259 could induce protective immunity against IAV/NTHi coinfection, we immunized mice i.n. with the purified 0259 protein adjuvanted with curdlan, a Th17-driving adjuvant (Fig. 6A)^56,57^. The unimmunized mice had only 30% survival after PR8/NT127 coinfection, whereas immunization with protein 0259 provided full protection (100% survival) against coinfection (Fig. 6B). Immunized mice lost less body weight and regained it faster than unimmunized mice (Fig. 6C). The pulmonary bacterial loads were significantly lower in the lungs of immunized mice than unimmunized mice at day 2 after NT127 infection (Fig. 6D). Protection in immunized mice correlated with increased NTHi-specific Th17 responses in the lung, as detected by ICS after *ex vivo* stimulation with protein 0259 (Supplementary Figs. S6A and S6B) or HK-NT127 (Figs. 6E and 6F). At day 12/7 post PR8/NT127, there were higher frequencies (Supplementary Fig. S6A and Fig. 6E) and total numbers (Supplementary Fig. S6B, and Fig. 6F) of IL-17A^+^ CD4^+^ T cells in immunized mice compared with unimmunized mice. These results show that immunization with protein 0259 plus curdlan induces protective immunity against respiratory coinfection correlating with enhanced Th17 responses.

To investigate if immunization with a single conserved protein among NTHi strains can provide broad protection against PR8/NTHi coinfection, immunized mice were challenged with PR8 and a different NTHi strain, 86-028NP. Compared with unimmunized mice, immunized mice had reduced mortality (Fig. 6G) and morbidity (Fig. 6H), lower bacterial burdens (Fig. 6I), and elevated Th17 responses in the lungs after PR8/86-028NP challenge (Figs. 6J and 6K; Supplementary Figs. S6C and S6D). These results show that immunization with a single protein 0259 can provide broad protection against secondary PR8/NTHi coinfection that correlated with enhanced pulmonary Th17 responses in the lung. Taken together, our data suggest the potential of this protein as a vaccine candidate against IAV/NTHi coinfection that is mediated by Th17 cells rather than traditional antibody-based immune mechanism of protection.

## DISCUSSION

Previous animal studies of IAV and bacterial coinfection have used relatively high doses of IAV and bacteria, with a clinical end point of death within 1 week after the bacterial infection^14–16^. However, as a result of annual influenza vaccination, many patients experience influenza virus infection with only mild symptoms^3–6^. It is not known if these episodes still render individuals susceptible to secondary bacterial pneumonia. Our study revealed that a mild or resolving influenza virus infection still induces susceptibility to bacterial multiplication in the lung, leading to severe pneumonia, lung pathology, and high mortality. This finding has important implications from a clinical and public health vaccination planning perspective since emphasis only on influenza virus vaccination may result in a patient population that contracts influenza virus and is still at risk of developing secondary bacterial pneumonia. In comparison to the widely used high pathogen doses coinfection model, which results in rapid mortality, our model of mild IAV infection more closely mimics clinic settings and enables the analysis of the adaptive immune response in surviving mice.

Th17 cells have an established role in protective immunity against bacterial and fungal infection^58,59^. The contribution of Th17 cells in lower respiratory infection with NTHi is not fully understood^47,60^. Our results, in agreement with our previous report^47^, showed that NTHi infection induced predominately NTHi-specific Th17 cells and a small percentage of Th1 cells, but IAV coinfection reduces the primary NTHi-specific Th17 cells in the lung and impairs pulmonary bacterial clearance compared with NTHi single infection. Previous reports observed that type I IFN, driven by primary viral infection, can inhibit IL-17-producing γδ T cells by limiting IL-23 expression^15,16^. Our data further support this concept of viral inhibition of the primary Th17 response against bacterial infection^61,62^. Our study examined if pre-existing memory Th17 cells can overcome viral-driven Th17 inhibition. We hypothesized that IAV infection could inhibit not only a primary Th17 response to NTHi but also a recall response of NTHi-specific memory Th17 cells, thus limiting protective capacity of memory Th17 cells against coinfection. Surprisingly, our data showed that, unlike in naïve mice, NTHi-specific memory Th17 cells in immunized mice were capable of mounting a Th17 recall response that reduced pulmonary NTHi burden and fully protected against morbidity and mortality after IAV/NTHi coinfection. Notably, the Th17 recall response during coinfection is less than the Th17 response during primary single infection. The bacterial burden in the primary single infection is higher (Fig. 1G) compared with bacteria burden in immune coinfected mice (Fig. 3F). These data suggest that antigen availability may contribute to the magnitude of the recall Th17 response. Our findings provide important rationales for pursuing a vaccine designed to elicit Th17 memory as a means to provide protective immunity against secondary bacterial pneumonia.

To date, the effector mechanisms used by memory Th17 cells to mediate protection remain unclear. It is possible that IL-17A signaling enhances the recruitment and activation of phagocytic innate immune cells that clear NTHi from the lung. Our results showed increased numbers of pulmonary alveolar macrophages at day 2 post challenge with coinfection in the immunized mice, whereas the absolute number of neutrophils and APCs were similar between unimmunized mice and immunized mice. Interestingly, MHC II expression was up-regulated on all these innate immune cells in the immunized group compared with unimmunized mice, which was positively correlated with stronger recall Th17 responses and rapid bacterial clearance. Studies have reported that macrophages and APCs expressing MHC II are capable of activating memory CD4^+^ Th17 cells^63^. Similarly, neutrophils can acquire the characteristics of APCs (MHC II expression) to present foreign antigens to CD4^+^ T cells and retain the ability to kill bacteria in mice^64,65^. Thus, after immunization, mice mounted protective Th17 recall responses in our study. In turn, the memory Th17 cells can recruit and activate inflammatory cells to clear bacteria through IL-17 signaling. Alveolar macrophages are the predominant resident phagocytes in the lung and are essential for clearance of low dose pneumococcal infection in the absence of the pronounced neutrophil recruitment that occurs in fulminant infection^66,67^. In agreement with these studies, the immunized mice in our study had more alveolar macrophages in the lungs upon coinfection, resulting in lower bacterial burdens compared with unimmunized mice. Future work will be needed to probe how IL-17A-producing CD4^+^ T cells activate the innate immune cells to kill bacteria in IAV/NTHi coinfected hosts. Alternatively, other Th17 effector molecules could be promoting lung healing by tissue regeneration or by limiting the production of tissue-damaging inflammatory cytokines. IL-22 expression from protective Th17 cells might contribute to the lung epithelial regeneration, for example^68^. Gaining a full appreciation of the protective repertoire of Th17 cells during influenza virus/bacterial coinfection will be important to guide vaccine developers in designing platforms that will generate protective Th17-based immunity.

Vaccination is the most effective method of preventing influenza virus infection and spread^69^. However, high genetic diversity and evolution of IAV facilitate escape from immune responses^70^. The currently available influenza virus vaccines are inadequate at protecting against emerging subtypes of influenza virus, allowing the persistence of flu complications, such as secondary bacterial pneumonia^37,38^. Our results show for the first time that a mild influenza virus infection can still lead to high susceptibility to secondary bacterial pneumonia, highlighting the importance of developing an effective vaccine against not only influenza virus but also secondary bacterial pathogens. The dysfunction of host immune responses during influenza virus infection is a primary cause of increased susceptibility to subsequent bacterial infection^19,71,72^. Insights from bacterial vaccine studies might provide protective strategies against IAV/bacterial coinfection. Our previous studies have shown that vaccine-induced antibodies recognize bacterial surface antigens and provide strain-specific protection^47,53^. In contrast, vaccine-induced memory Th17 cells provide serotype-independent cross-protection by recognizing conserved intracellular bacterial protein antigens^73–75^. Furthermore, immunization with a conserved NTHi protein induced memory Th17 cells that are protective against diverse NTHi strains. These results support a broadly protective role of memory Th17 cells during IAV/NTHi coinfection and suggest a promising approach to develop effective vaccines against the deadly secondary bacterial pneumonia associated with IAV infection.

## MATERIALS AND METHODS

### Animals

Female C57BL/6 mice (6–8 weeks old) were purchased from National Cancer Institute (Fredericksburg, Maryland, USA). The B-cell–deficient (μMT^−/−^) mice (8 weeks old) were obtained from Jackson Laboratory. All animal experiments were performed in accordance with Institutional Animal Care and Use Committee approved protocols.

### Pathogens and infections

NTHi strains NT127 and 86-028NP were grown in brain heart infusion (BHI) broth supplemented with 2.0% (v/v) Fildes enrichment (BD, Franklin Lakes, NJ, USA) and 10 μg/ml nicotinamide adenine dinucleotide (NAD) (Sigma, St. Louis, MO, USA) or on supplemented BHI (sBHI) agar plates as described^51^. *S. pneumoniae* strain TIGR4 (T4, serotype 4) was cultured in tryptic soy broth or agar plates, as previously described^53^. For the preparation of heat-killed (HK), the bacterial suspensions were heated at 65 °C for 30 minutes and plated to ensure 100% bacterial killing. Mouse-adapted influenza A/Puerto Rico/8/34 (PR8; H1N1) virus strain was propagated in specific pathogen-free fertile chicken eggs, as previously described^76^.

Before being inoculated with virus and/or bacteria intranasally (i.n.), mice were anesthetized by intraperitoneal injection with 100 μl ketamine/xylazine (100 mg/3.8 mg/kg). The mice were infected with 200 or 50 tissue culture ID_50_ (TCID_50_) of PR8. After 5 days, the experimental animals were inoculated with 30 μl suspensions of NTHi (10^1^-10^8^ CFU). The control animals were inoculated with PBS.

For immunization using live NTHi or protein 0259 plus curdlan (Sigma, St. Louis, MO, USA), the mice were immunized i.n. with a 30 μl suspension of NTHi (10^8^ CFU) or protein 0259 (10 μg) plus the adjuvant curdlan (200 μg). On day 21 after immunization, while anesthetized, the mice were challenged i.n. with 30 μl of 50 TCID50 of PR8 and with 30 μl suspensions of NTHi (10^8^ CFU), as indicated. On day 2 and day 4 after the challenge, NTHi CFUs in the bronchoalveolar lavage (BAL) fluids and lung homogenates were determined^53,77^.

The mice were observed for clinical signs of morbidity by monitoring body weight, atrial oxygen saturation, breath rate, and heart rate parameters daily using the MouseOx Small Animal Vital Signs Monitor Mouse Ox plus (Starr Life Sciences, Oakmont, PA, USA) on conscious (nonanesthetized) mice attaching a collar clip after the pathogen infection. The samples of BAL fluid, lung homogenates, and heparinized blood were prepared, as described^53^, and 10 μl of serially dilutions were plated in triplicate. The limit of detection for bacteria in the lavage, blood, or lung homogenate was 10 CFU/ml. The viral loads in lung homogenates were determined by polymerase RNA of PR8 through quantitative real-time reverse transcription polymerase chain reaction and were normalized by the housekeeping gene HPRT of mouse, as described^78,79^.

### Intracellular cytokine staining and flow cytometry

The lymphocytes from lungs were isolated and stained, as previously described^80,81^. For intracellular staining, cells were stimulated with HK bacteria (65°C for 30 minutes) at indicated multiplicity of infection for 16 hours, with golgi plug/stop added for the last 4 hours. The cells were then stained as described in our previous study^47,53^. LSRII (BD, Franklin Lakes, NJ, USA) was used for acquiring samples. Flow data were analyzed by FlowJo version10.4 (FlowJo LLC, Ashland, OR, USA).

### *In vivo* antibody treatment

IL-17A neutralization was achieved as previously described, with slight modification^82–84^. Mice received 1000 μg anti-IL-17A antibody (Clone 17F3, BioXcell) intraperitoneally on day −1, 0, 1 of NTHi challenge and 80 μg i.n. of anti-IL-17A antibody on day 0 of challenge. The BAL fluid was collected on day 2 after infection. The neutralizing efficiency was verified by enzyme-linked immunosorbent assay (ELISA).

### Adoptive transfer experiments

For serum transfer, each recipient mouse was passively transferred with 200 μl sera from immunized mice by tail vein injection. The total CD4^+^ T cells were isolated from the lung of immunized mice using MACS CD4^+^ T Cell Isolation Kit (Miltenyi Biotec, Bergisch Gladbach, NRW, Germany) and were confirmed the purity (>95%) by flow cytometry. The purified CD4^+^ T cells (2–4 × 10^6^) were transferred intravenously into recipient mice 1 day before 86-028NP infection.

### ELISA

The diluted HK bacteria (approximately 3 × 10^8^ CFU) were coated onto 96-well plates at 37 °C for 1 hour. The wells were blocked with 2% nonfat milk in PBS, and three-fold serial dilutions of pooled mouse sera were applied to the wells, in duplicate. HK bacteria-coated plates were incubated with sera for 1 hour at 37°C. Antibody binding was detected with goat anti-mouse IgG-HRP (H + L). For the ELISA assay readout, TMB peroxidase substrate (BioLegend, San Diego, CA, USA) was used according to the manufacturer’s instructions. The optical densities were read at 450 nm with a microplate reader (SpectraMax 190 Absorbance Microplate Reader). Mouse IL-17A or IFN-γ MAX standard sets (BioLegend, San Diego, CA, USA) were used for detection of IL-17A or IFN-γ from BAL fluid according to the manufacturer’s protocols.

### Histology

Lung sections were prepared as described previously^85^. The sections were processed and stained by the Cardiology Histology Core and Cancer Histology Core at the University of Pennsylvania. The degree of inflammation was scored by a blinded pathologist and was given a score of 0–3 as described^86^: 0, no inflammation; 1, a small number of inflammatory cells; 2, a medium number of inflammatory cells; and 3, a large number of inflammatory cells.

### Expression and purification of recombinant nontypeable *Haemophilus influenzae* 0259 protein from *Escherichia coli*

NT127 gene HIAG_0259 cloned in the pET28a expression vector was described in our previous study^47^. This *Escherichia coli*-containing expression vector was grown in Luria-Bertani (LB) broth in the presence of 0.1 mM IPTG and cultured for 4 hours at 30 °C. Protein 0259 was purified over Ni columns as previously described^47^ and was analyzed by SDS/PAGE with Coomassie staining to confirm the purity.

### Statistical analyses

Unpaired, one-tailed Student’s t test was used to calculate statistical significance between two groups and a one-way analysis of variance for multiple group comparison, followed by Bonferroni correction, unless stated otherwise. Survival curves were analyzed using Log-Rank analysis *p* values as follows: **** *p* < 0.0001; *** *p* < 0.001; ** *p* < 0.01; * *p* < 0.05 and ^ns^*p* > 0.05. A *p* value ≤ 0.05 was considered significant.

## Supplementary Material

1

## Data Availability

All data generated or analyzed during this study are available from the corresponding author on reasonable request.
