# Supplementary material for: Antigen-specific memory Th17 cells promote cross-protection against nontypeable Haemophilus influenzae after mild influenza A virus infection
Source: Mucosal Immunol. Author manuscript; Available in PMC 2023 Jul 13. (PMC10343962; doi:10.1016/j.mucimm.2023.01.007)
Supplement: 1 [file NIHMS1909475-supplement-1.pdf]

Fig S1

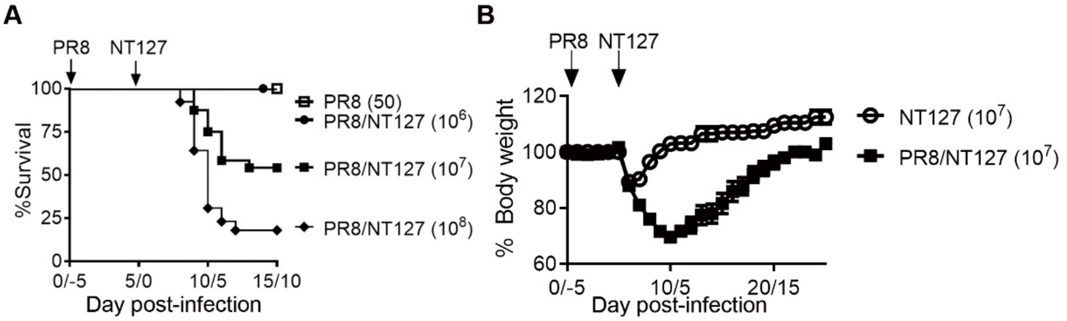

Fig. S1. Influenza virus infection increases susceptibility to NTHi infection. (A) Mice were first infected with 50 $TCID_{50}$ of PR8. After 5 days, the mice were infected with $10^{6\text{-}8}$ CFU NT127. Survival rates and (B) body weight loss were measured daily. Data is representative of three independent experiments with 9-10 mice in each group. Data is shown as mean ± SEM.

Fig S2

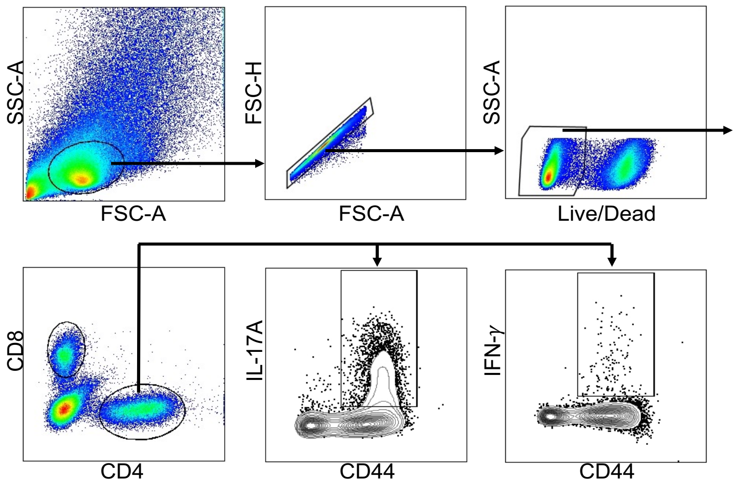

Fig. S2. Gating strategy used to identify IL-17A⁺CD4⁺ and IFN-γ⁺CD4⁺ T cell subsets in mouse lung. Th17 and Th1 cells were identified by IL-17A and IFN-γ cytokine staining in $CD4^+$ T cells after exclusion of debris, doublets and dead cells.

Fig S3

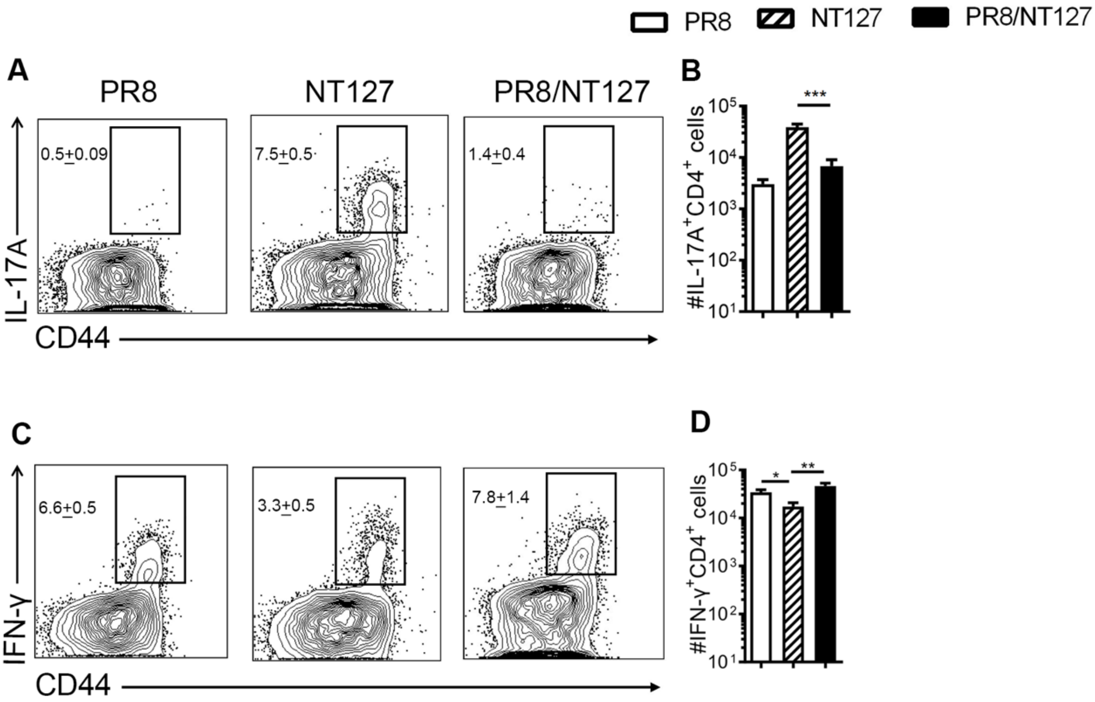

Fig. S3. Influenza virus infection suppresses total Th17 response during co-infection in the lung. (A-D) IL-17A and IFN-γ production by CD4⁺ T cells after *ex vivo* stimulation with PMA/Ionomycin in cell isolated from the lungs of PR8-, NT127- and PR8/NT127- infected mice. Data are expressed as the mean value ± SEM of 5-10 mice/group, representative of three independent experiments. ***P < 0.001; **P < 0.01; *P < 0.05.

Fig S4

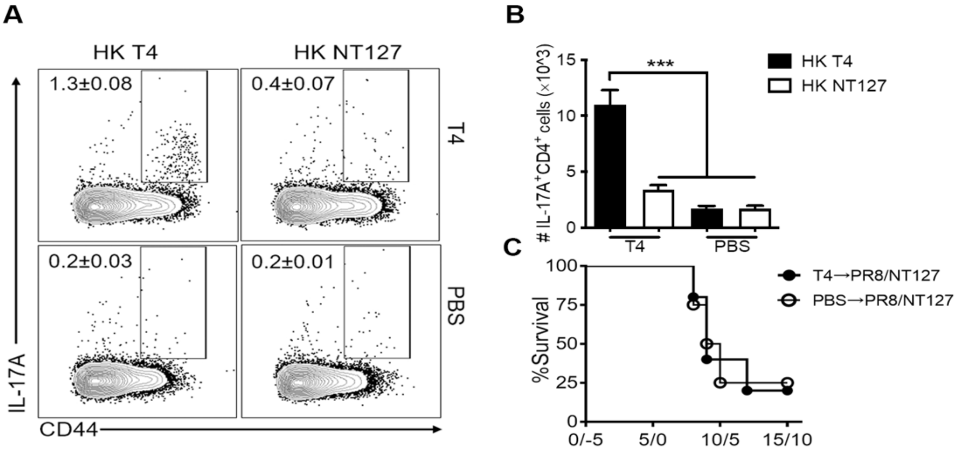

Fig. S4. *S. pneumoniae*-specific memory Th17 cells provide no protection against PR8/NT127 co-infection. Mice were intranasally immunized with *S. pneumoniae* (T4 strain, 10^6 CFUs) or PBS. On day 21, lymphocytes from the lung were isolated and then were stimulated with heat-killed (HK) T4 or HK NT127. (A) Frequency and (B) absolute number of IL-17A producing $CD4^+$ T cells in the lung (n=3-5 mice per group). (C) Immunized mice were challenged with PR8/NT127 on day 21 after immunization. Survival rates were recorded after co-infection (n=8-10 mice per group). Data are representative of two independent experiments. Error bars = means ± SEM. ***$P < 0.001$.

Fig S5

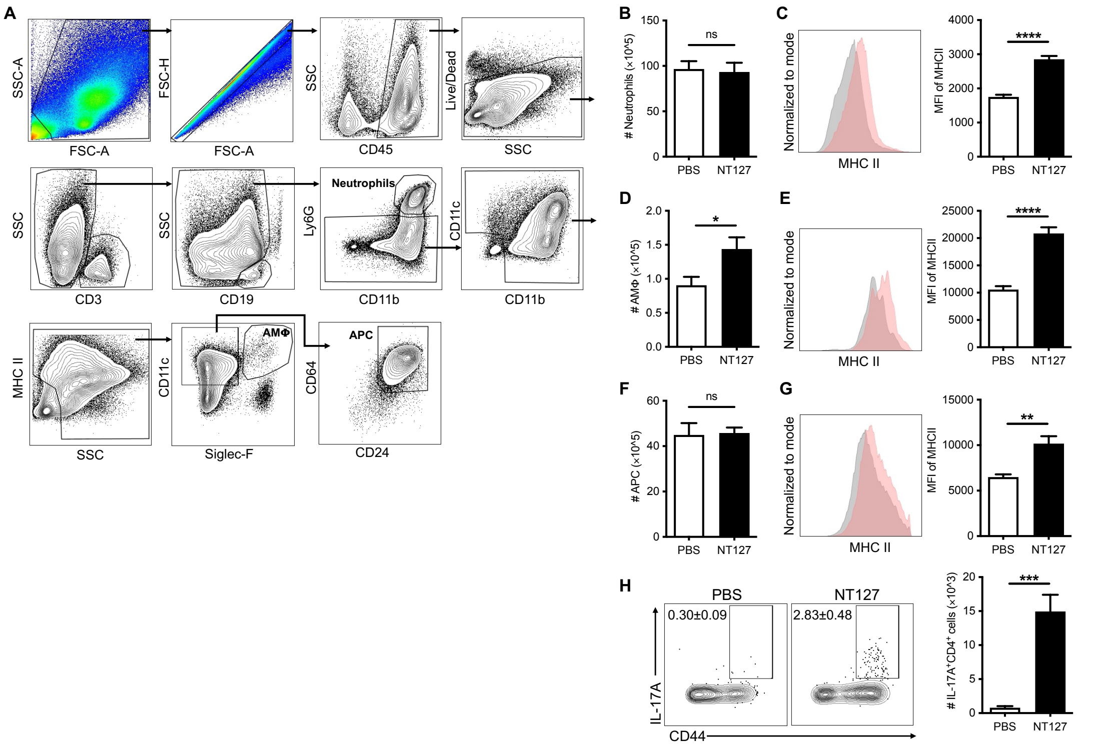

Fig. S5. The pulmonary residential innate immune cells and recall Th17 responses are elevated in NT127 immunized mice. (A) Gating strategy used to identify neutrophils, alveolar macrophages (AM**Φ**) and antigen-presenting cells (APCs, $CD11c^{+}CD24^{+}CD64^{+}Siglec\text{-}F^{neg}$) in mouse lung. Total numbers and MHC II expression of neutrophils (B, C), AM**Φ** (D, E) and APCs (F, G) at day 7/2 post PR8/86-028NP co-infection in the lung of PBS- (grey) and NT127- (red) immunized mice. (H) Recall Th17 responses ($IL\text{-}17A^{+}CD4^{+}$ T cells) at day 7/2 post co-infection in the lung of PBS and NT127 immunized mice (n=4-5 mice per group). Data are representative of two independent experiments. Error bars = means ± SEM. *****P* < 0.0001; ****P* < 0.001; ***P* < 0.01; **P* < 0.05; ns, not significant.

Fig S6

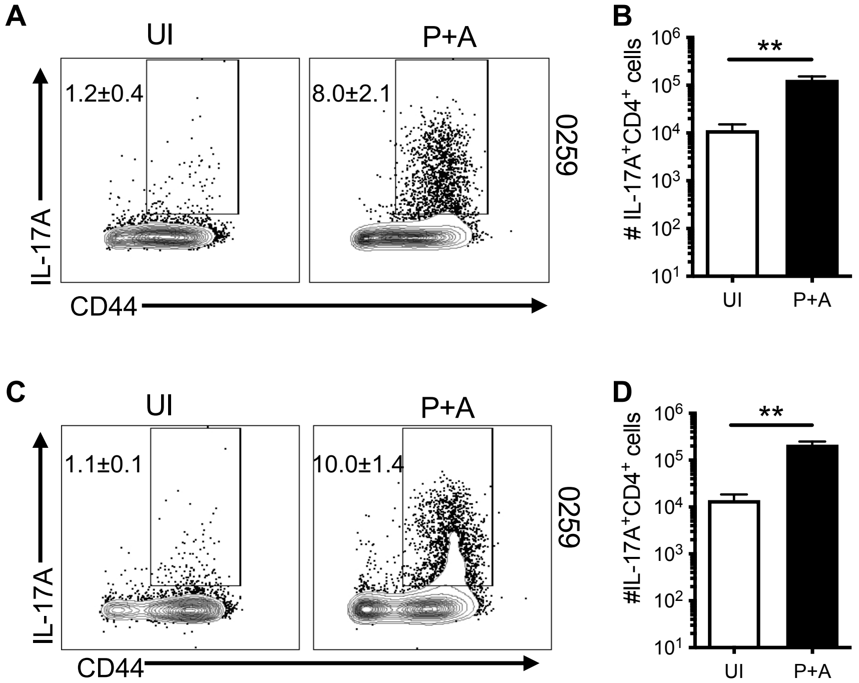

Fig. S6. Immunization with an identified Th17 antigen induces strong antigen-specific Th17 responses. B6 mice were immunized intranasally with purified protein 0259 plus the curdlan adjuvant (P+A), three times at one-week intervals. Unimmunized (UI) mice were included as control. Three weeks post final immunization, these mice were challenged intranasally by PR8 and NTHi. (A) Frequency and (B) absolute number of pulmonary IL-17A producing $CD4^+$ T cells isolated from the lung on day 7 after homogenous NT127 infection following *ex vivo* stimulation with protein 0259 (n=5 mice per group). (C) Frequency and (D) absolute number of IL-17A producing $CD4^+$ T cells isolated from the lung on day 7 after heterologous 86-028NP infection following *ex vivo* stimulation with protein 0259 (n=3-5 mice per group). Data are representative of two independent experiments. Error bars = means ± SEM. *****P* < 0.0001; ****P* < 0.001; ***P* < 0.01; **P* < 0.05.

Table S1: Antibodies for flow cytometry

Antibody	Clone	Fluorochrome	Source
T cell response panel			
CD4	RM4-5	Qdot605	Invitrogen
CD8	5H10	Pac Blue	Invitrogen
CD44	IM7	PerCP-Cy5.5	eBioscience
IL-17A	TC11-18H10	PE	BD Pharmingen
IFN-γ	XMG1.2	APC	eBioscience
Innate cell panel			
CD45	30-F11	APC-Cy7	BioLegend
CD3	145-2C11	PE-Cy5	BioLegend
CD19	6D5	BV785	BioLegend
Ly6G	1A8	AF700	BioLegend
CD11b	M1/70	PerCP-Cy5.5	BD Biosciences
CD11c	N418	APC	eBioscience
MHC II	M5/114.15.2	eF450	eBioscience
Siglec F	E50-2440	PE	BD Pharmingen
CD24	M1/69	BV711	BD Biosciences
CD64	X54-5/7.1	BV650	BD Biosciences
Other			
Live/Dead Fixable Dead Cell Stain		Aqua	Invitrogen
